# 1074. Surveillance of SARS-CoV-2 variants of concern (VOC) in hospital wastewater (WW) and its correlation with hospitalized cases of COVID-19 and the occurrence of outbreaks

**DOI:** 10.1093/ofid/ofac492.915

**Published:** 2022-12-15

**Authors:** Nicole Acosta, Maria Bautista Chavarriaga, Barbara Jean M Waddell, Janine McCalder, Kristine Du, Puja Pradhan, Navid Sedaghat, Chloe Papparis, Jianwei Chen, Jennifer Van Doorn, Kevin Xiang, Leslie Chan, Laura Vivas, Kashtin Low, Xuewen Lu, Thierry Chekouo, Xiaotian Dai, Jason Cabaj, Steve Hrudey, Srijak Bhatnagar, Norma J Ruecker, Gopal Achari, M Cathryn Ryan, Rhonda Clark, Craig Pearce, Joe Harrison, Jon Meddings, Jenine Leal, Bayan Missaghi, Jamil Kanji, Oscar Larios, Elissa Rennert May, Joseph Kim, Xiao-Li Pang, Bonita Lee, Kevin Frankowski, John M Conly, Casey R J Hubert, Michael Parkins

**Affiliations:** University of Calgary, Calgary, Alberta, Canada; University of Calgary, Calgary, Alberta, Canada; University of Calgary, Calgary, Alberta, Canada; University of Calgary, Calgary, Alberta, Canada; University of Calgary, Calgary, Alberta, Canada; University of Calgary, Calgary, Alberta, Canada; University of Calgary, Calgary, Alberta, Canada; University of Calgary, Calgary, Alberta, Canada; University of Calgary, Calgary, Alberta, Canada; University of Calgary, Calgary, Alberta, Canada; University of Calgary, Calgary, Alberta, Canada; University of Calgary, Calgary, Alberta, Canada; University of Calgary, Calgary, Alberta, Canada; University of Calgary, Calgary, Alberta, Canada; University of Calgary, Calgary, Alberta, Canada; University of Calgary, Calgary, Alberta, Canada; University of Calgary, Calgary, Alberta, Canada; University of Calgary, Calgary, Alberta, Canada; University of Alberta, Edmonton, Alberta, Canada; University of Calgary, Calgary, Alberta, Canada; Calgary City, Calgary, Alberta, Canada; University of Calgary, Calgary, Alberta, Canada; University of Calgary, Calgary, Alberta, Canada; University of Calgary, Calgary, Alberta, Canada; University of Calgary, Calgary, Alberta, Canada; University of Calgary, Calgary, Alberta, Canada; University of Calgary, Calgary, Alberta, Canada; University of Calgary, Calgary, Alberta, Canada; University of Calgary, Calgary, Alberta, Canada; University of Alberta, Edmonton, Alberta, Canada; University of Calgary, Calgary, Alberta, Canada; University of Calgary, Calgary, Alberta, Canada; University of Calgary, Calgary, Alberta, Canada; University of Alberta, Edmonton, Alberta, Canada; University of Alberta, Edmonton, Alberta, Canada; University of Calgary, Calgary, Alberta, Canada; University of Calgary, Calgary, Alberta, Canada; University of Calgary, Calgary, Alberta, Canada; University of Calgary, Calgary, Alberta, Canada

## Abstract

**Background:**

WW surveillance enables real time monitoring of SARS-CoV-2 burden in defined sewer catchment areas. Here, we assessed the occurrence of total, Delta and Omicron SARS-CoV-2 RNA in sewage from three tertiary-care hospitals in Calgary, Canada.

**Methods:**

Nucleic acid was extracted from hospital (H) WW using the 4S-silica column method. H-1 and H-2 were assessed via a single autosampler whereas H-3 required three separate monitoring devices (a-c). SARS-CoV-2 RNA was quantified using two RT-qPCR approaches targeting the nucleocapsid gene; N1 and N200 assays, and the R203K/G204R and R203M mutations. Assays were positive if Cq< 40. Cross-correlation function analyses (CCF) was performed to determine the time-lagged relationships between WW signal and clinical cases. SARS-CoV-2 RNA abundance was compared to total hospitalized cases, nosocomial-acquired cases, and outbreaks. Statistical analyses were conducted using R.

**Results:**

Ninety-six percent (188/196) of WW samples collected between Aug/21-Jan/22 were positive for SARS-CoV-2. Omicron rapidly supplanted Delta by mid-December and this correlated with lack of Delta-associated H-transmissions during a period of frequent outbreaks. The CCF analysis showed a positive autocorrelation between the RNA concentration and total cases, where the most dominant cross correlations occurred between -3 and 0 lags (weeks) (Cross-correlation values: 0.75, 0.579, 0.608, 0.528 and 0.746 for H-1, H-2, H-3a, H-3b and H-3c; respectively). VOC-specific assessments showed this positive association only to hold true for Omicron across all hospitals (cross-correlation occurred at lags -2 and 0, CFF value range between 0.648 -0.984). We observed a significant difference in median copies/ml SARS-CoV-2 N-1 between outbreak-free periods vs outbreaks for H-1 (46 [IQR: 11-150] vs 742 [IQR: 162-1176], P< 0.0001), H-2 (24 [IQR: 6-167] vs 214 [IQR: 57-560], P=0.009) and H-3c (2.32 [IQR: 0-19] vs 129 [IQR: 14-274], P=0.001).

**Conclusion:**

WW surveillance is a powerful tool for early detection and monitoring of circulating SARS-CoV-2 VOCs. Total SARS-CoV-2 and VOC-specific WW signal correlated with hospitalized prevalent cases of COVID-19 and outbreak occurrence.

**Disclosures:**

**All Authors**: No reported disclosures.

